# Home Blood Pressure Self-monitoring plus Self-titration of Antihypertensive Medication for Poorly Controlled Hypertension in Primary Care: the ADAMPA Randomized Clinical Trial

**DOI:** 10.1007/s11606-022-07791-z

**Published:** 2022-10-11

**Authors:** Patricia Martínez-Ibáñez, Irene Marco-Moreno, Salvador Peiró, Lucia Martínez-Ibáñez, Ignacio Barreira-Franch, Laura Bellot-Pujalte, Eugenia Avelino-Hidalgo, Marina Escrig-Veses, María Bóveda-García, Mercedes Calleja-del-Ser, Andreu Ferrero-Gregori, Adina A. Iftimi, Isabel Hurtado, Aníbal García-Sempere, Clara L Rodríguez-Bernal, Margarita Giménez-Loreiro, Gabriel Sanfélix-Gimeno, José Sanfélix-Genovés, the ADAMPA research group, J Abad Carrasco, MV Agudo Escagüés, E Avelino-Hidalgo, I Barreira-Franch, RM Bartual Penella, L Bellot-Pujalte, M Bóveda-García, M Calleja Del Ser, R Carrión Villanueva, A Costa Alcaraz, I Cristófol López, M Escrig-Veses, A Ferrero-Gregori, A García-Sempere, M Giménez-Loreiro, R González Candelas, R González Espadas, L González Luján, V Gosalbes, E Guinot Martínez, I Hurtado, AA Iftimi, EL López Torres, I Marco-Moreno, P Martinez-Ibañez, L Martinez Ibañez, S Molla Llosa, V Moreno Comins, M Moreno Prat, J Navarro-Pérez, S Peiró, MJ Puchades Company, A Ramos García, P Ramos Ruiz, P Roca Navarro, C Rodríguez-Bernal, R Saiz Rodriguez, JL Salanova Chilet, J Sanfélix-Genovés, G Sanfélix-Gimeno, A Tchang Sanchez, F Torres Asensi, R Uribes Fillol, C Valle García, M Villar Ruiz

**Affiliations:** 1 INCLIVA Health Research Institute, Valencia, Spain; 2 Health Services Research Unit, FISABIO, Catalunya Av. 21, 46020 Valencia, Spain; 3 Department of Statistics and Operations Research, Universidad de Valencia, Valencia, Spain; 4 Network for Health Services Research in Chronic Diseases (REDISSEC), Valencia, Spain; 5 Research Network On Chronicity, Primary Care, and Health Promotion (RICAPPS), Valencia, Spain

**Keywords:** patient empowerment, blood pressure self-monitoring, blood pressure self-management, primary care, randomized clinical trial

## Abstract

**Background:**

Patient empowerment through pharmacological self-management is a common strategy in some chronic diseases such as diabetes, but it is rarely used for controlling blood pressure.

**Objective:**

This study aimed to assess self-monitoring plus self-titration of antihypertensive medication versus usual care for reducing systolic blood pressure (SBP) at 12 months in poorly controlled hypertensive patients.

**Design:**

The ADAMPA study was a pragmatic, controlled, randomized, non-masked clinical trial with two parallel arms in Valencia, Spain.

**Participants:**

Hypertensive patients older than 40 years, with SBP over 145 mmHg and/or diastolic blood pressure (DBP) over 90 mmHg, were recruited from July 2017 to June 2018.

**Intervention:**

Participants were randomized 1:1 to usual care versus an individualized, pre-arranged plan based on self-monitoring plus self-titration.

**Main Measure:**

The primary outcome was the adjusted mean difference (AMD) in SBP between groups at 12 months.

**Key Results:**

Primary outcome data were available for 312 patients (intervention *n*=156, control *n*=156) of the 366 who were initially recruited. The AMD in SBP at 12 months (main analysis) was −2.9 mmHg (95% CI, −5.9 to 0.1, *p*=0.061), while the AMD in DBP was −1.9 mmHg (95% CI, −3.7 to 0.0, *p*=0.052). The results of the subgroup analysis were consistent with these for the main outcome measures. More patients in the intervention group achieved good blood pressure control (<140/90 mmHg) at 12 months than in the control group (55.8% vs 42.3%, difference 13.5%, 95% CI, 2.5 to 24.5%, *p*=0.017). At 12 months, no differences were observed in behavior, quality of life, use of health services, or adverse events.

**Conclusion:**

Self-monitoring plus self-titration of antihypertensive medication based on an individualized pre-arranged plan used in primary care may be a promising strategy for reducing blood pressure at 12 months compared to usual care, without increasing healthcare utilization or adverse events.

**Trial Registration:**

EudraCT, number 2016-003986-25 (registered 17 March 2017) and clinicaltrials.gov, NCT03242785.

**Supplementary Information:**

The online version contains supplementary material available at 10.1007/s11606-022-07791-z.

## BACKGROUND

Cardiovascular diseases are the main cause of disability and premature death worldwide,^1–3^ and high blood pressure (BP) is the main modifiable risk factor.^4^ While strong evidence supports the benefits of blood pressure control for avoiding cardiovascular complications,^5–8^ several studies suggest that, despite recent improvements, a significant proportion of hypertensive patients remains poorly controlled.^2, 5, 9–11^ Therefore, the development and assessment of new interventions that can potentially improve BP control are of outstanding relevance, especially when these are inexpensive and based in the primary care setting, where diagnosis, treatment, and monitoring of hypertension usually take place.

Among the array of interventions proposed to improve BP control,^12–15^ one prominent strategy is based on home blood pressure monitoring (HBPM),^13, 16–19^ which may include telemonitoring^20–23^ and/or patient/health professional treatment adjustment.^24–27^ However, these interventions have yielded disparate results. Current BP monitoring devices, which have lower prices and are easy to use, have facilitated the widespread use of HBPM, with some advantages over clinic-based monitoring.^28^ While HBPM alone does not seem to be associated with better BP control rates,^13, 16, 17, 19, 25^ in combination with other co-interventions, it results in a moderate but clinically significant reduction in BP values.^18, 19, 23–27^

Patient empowerment through pharmacological self-management is a common strategy in some chronic diseases such as diabetes, but it is rarely used for controlling BP. When it is, it is done with very different degrees of intensity. Often, rather than a significant increase in patient empowerment (especially with regard to treatment self-adjustment), strategies involve greater monitoring and/or use of human resources (health and non-health-related) or additional technologies, such as telemonitoring.^24–27^

HBPM interventions with self-monitoring of blood pressure plus different strategies of self-adjustment of hypertensive medication might contribute to better hypertension control and offer promising evidence of effectiveness,^29^ with no increase in side effects,^23, 26^ an acceptable cost-effectiveness ratio,^30, 31^ and satisfactory acceptance by patients^32^ and professionals.^33, 34^ However, evidence is limited to few trials^24–27, 35^ with heterogeneous samples, intervention components, and different levels of patient empowerment (through self-management).^29^

The aim of this trial was to evaluate the effectiveness of an intervention including self-monitoring of blood pressure plus self-titration of antihypertensive medication (based on an individualized pre-arranged plan) and educational components versus usual care (also with educational components) for reducing blood pressure in poorly controlled hypertensive patients in the primary care setting.

## METHODS

### Study Design

The ADAMPA study is a pragmatic, controlled, randomized, non-masked clinical trial with two parallel arms. It took place in a Valencia health district (Spain) and involved 36 family doctors (27 of whom recruited patients from 15 primary healthcare centers). The study protocol was published elsewhere.^36^

### Setting

The ADAMPA study took place in one health district of the Valencia health system, serving a population of 345,000 inhabitants. This district is part of an extensive network of public hospitals and primary healthcare centers, part of the Spanish National Health System, which provides virtually universal healthcare that is free at the point of service (except for some co-payments for out-of-hospital medication). Recruitment took place from July 2017 to June 2018, with a follow-up of 12 months.

### Participants

Patients with a diagnosis of hypertension in their electronic medical record, aged 40 years and over, with uncontrolled hypertension (mean BP reading on the reference arm of systolic BP (SBP) > 145 mmHg or diastolic BP (DBP) > 90 mmHg on the baseline examination) and voluntarily agreeing to join the study were eligible for inclusion (see exclusion criteria in eMethods in the Appendix).

### Randomization and Blinding

**Figure 1:**
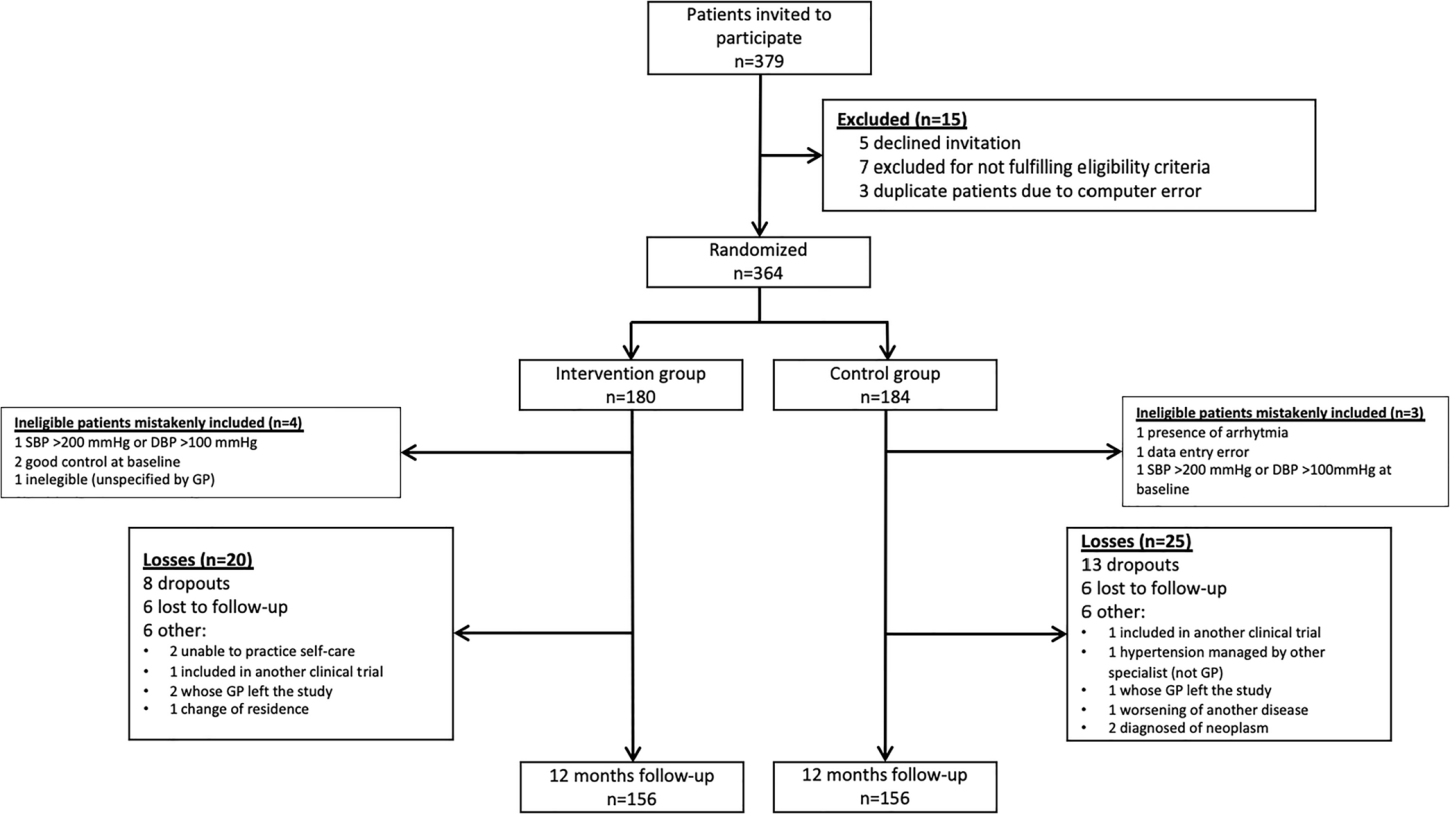
ADAMPA patient flow chart.

Family doctors recruited potentially eligible patients, performed a preliminary examination, and obtained written informed consent from participants. The sample was randomized in a 1:1 ratio using a centralized online randomization system to usual care or self-management. A minimization strategy^37^ was used to consider age, gender, SBP > 160mmHg, and comorbidities (diabetes, cardiovascular disease, stroke, and chronic kidney disease). A comprehensive baseline examination was scheduled after randomization; a few patients either dropped out beforehand or were excluded because the examination revealed that they were ineligible and had mistakenly been randomized (Fig. 1).

### Intervention

In the intervention group, the family doctor established with each patient a reference arm to measure blood pressure and an individualized BP target. These individualized goals were decided by the physician in conjunction with the patient, who received the European Society of Hypertension (ESH) and the European Society of Cardiology’s (ESC) 2013 guidelines for the management of hypertension^38^ (in force at the time of recruitment) and then the 2018 update,^39^ released during the study. They instructed participants on how to proceed according to their BP measurements using a color traffic light system (very high or very low readings required a visit to the family physician’s practice, while readings above target but below high limits required self-adjustment without the need to contact any health professional, see Figure S1). Participants also received written instructions with the medication self-adjustment plan (Figure S2). A member of the research team recorded additional baseline data (not recorded at the moment of inclusion), provided patients with a validated home blood pressure monitor (Omron M3 model HEM-7131-E), and trained them in their use. Participants also received an “Intervention group booklet” containing information and basic recommendations for improving BP control, information to correctly measure BP at home, BP targets, instructions on how to proceed according to their BP, and monthly BP record sheets for a 6-month period. They were asked to record their BP twice a day for the first 7 days of each month, once in the morning and once in the evening, plus all contacts with healthcare professionals for BP-related reasons (by phone, regular or urgent consultation at the healthcare center, or hospital visits) during that follow-up period. At the 6-month follow-up visit, participants received new monthly registration sheets to record their BP values up to the 12-month time point. To reach their target BP, each patient was given a self-management plan to adjust medication when BP readings were above target, with instructions for increasing the dose or adding new medication if necessary (Figure S2). The design of the therapeutic plan was at the discretion of the family doctor.

Participants proceeded to self-adjust, without any additional contact with their family doctor, other health workers, or coaches, when SBP or DBP was above the target for four or more days of the first week of the month. The self-adjustment had to be followed continuously until the following appointment with the doctor, which was 3 weeks after beginning the self-adjusted regimen (entailing strong patient empowerment). At the subsequent follow-up, a new tailored self-management plan was provided. Apart from the intervention of self-adjustment, all patients received routine hypertension care with appointments and medication changes following the family doctor’s criteria in the context of routine clinical practice. All relevant concomitant care within the usual clinical practice was at the discretion of the attending family physician.

Participants in the control group were informed by their family doctor that they would continue their usual care. Next, a member of the research team recorded additional baseline data (not recorded at the moment of inclusion) and provided patients with the “Control group booklet,” containing general information and basic recommendations for improving BP control, as well as monthly registration sheets to record BP-related healthcare encounters during the two consecutive follow-up periods (at 6 months and 12 months from baseline). The control group received routine hypertension care with appointments and medication changes following the family doctor’s criteria in the context of routine clinical practice. As in the intervention group, all relevant concomitant care within the usual clinical practice was at the discretion of the family doctor.

### Outcomes

The primary outcome of the study was the adjusted mean difference (AMD) in systolic blood pressure between the intervention and control groups at 12 months. At baseline (before randomization) and follow-up visits at the primary care health center, at least two BP readings were taken in a seated position, at 1- to 2-min intervals. If the first two readings were substantially different (at least 10 mmHg, as recommended by the ESH/ESC guidelines^38^), an additional reading was taken, and the mean value was calculated from the two readings considered valid. These readings were taken by the family physician using a validated home blood pressure monitor (Omron M3 model HEM-7131-E).

Secondary outcomes included the following: (1) AMD in DBP between the intervention and control groups at 12 months, (2) difference in the percentage of patients with optimal control between groups at 12 months (general recommendation and by age range, Table S1), (3) score obtained in the EuroQol-5D quality of life questionnaire at 12 months, (4) behavioral changes (smoking, exercise, body weight) between baseline and 12 months between the intervention and control groups, (5) use of health services for hypertension at 12 months, and (6) appearance of adverse events during the study period.

### Sample Size

A sample size of 382 patients was estimated in order to have 90% power to detect a 5 mmHg (SD 15 mmHg) difference in SBP between groups (primary outcome) with a two-tailed contrast and an alpha error of 0.05. This figure represents a clinically relevant difference based on previous trials.^23, 25, 26^ We increased this figure to 458 participants (20% increase) to account for possible dropouts and loss to follow-up. Recruitment was interrupted after 1 year for reasons unrelated to the study that affected some research family physicians (structural changes in the health department, involving transfer of some research doctors to other healthcare centers with assignment of new patients that did not guarantee the adequate recruitment and follow-up). Finally, 366 patients were randomized, and 312 completed follow-up, resulting in 84% power for the main analysis.

### Statistical Analysis

The analysis was performed on an intention-to-treat basis. A descriptive analysis of the groups’ baseline characteristics was performed using the *χ*^2^ test for categorical variables and Student’s *t*-test for continuous variables. We then estimated crude differences in SBP and DBP readings, with their corresponding 95% confidence intervals (CIs), between baseline and 12 months’ follow-up, as well as the MDs and 95% CIs between groups in SBP and DBP at 12 months. As pre-specified in the protocol, a linear mixed-effects analysis was performed to compare SBP between groups at 12 months. We adjusted for gender, age, baseline SBP, obesity, and diabetes as fixed effects, and for family physician as a random effect. Visual inspection of the residual plots did not show any major deviations from homoscedasticity or normality. The analyses for the DBP (secondary outcome) were carried out using similar techniques. We also estimated the proportion of patients with optimal control at 12 months using the overall recommendations (BP < 140/90 mmHg) and the specific ones for age groups established in the 2018 ESH/ESC guidelines,^39^ as well as the difference in proportions between groups.

Stratified analyses of between-group MD in SBP at 12 months, with their corresponding 95% CIs, were estimated according to gender, age (40 to 64 years, 65 to 79 years, and ≥ 80 years), baseline SBP (< 160 mmHg vs ≥ 160 mmHg), diabetes, other comorbidities (cerebrovascular disease, peripheral artery disease, chronic kidney disease, angina, or acute myocardial infarction), diabetes plus baseline SBP threshold, obesity, overweight/obesity, and obesity plus baseline SBP threshold. Differences between groups at 12 months’ follow-up were estimated for several secondary behavioral outcomes (smoking, obesity, sedentarism), health-related quality of life, the use of health services, and the incidence of adverse events during the study period.

In addition, we compared pharmacological treatments between groups at the 12-month visit to provide additional information on the differential therapeutic management between groups.

Two-sided *p* values of less than 0.05 were considered significant. Analyses were performed using STATA version 14 and R version 3.6.0.

## RESULTS

Of the 366 participants initially recruited, 312 (85.2%; intervention *n*=156; control *n*=156) attended the 12-month follow-up visit and contributed complete data for the primary outcome. Seven (1.9%) were excluded because they had been mistakenly included, 23 (6.3%) dropped out of the study, 12 (3.3%) were lost to follow-up, and 12 (3.3%) were excluded for other reasons (inability to practice blood pressure self-care, change of residence, primary care doctor left study) (Fig. 1).

Participants’ baseline sociodemographic and clinical characteristics were very similar between groups, except for sedentarism, which was more common in the intervention group (Table 1). Overall, 46.8% were men, the mean age was 64.4 years old, and mean baseline SBP was 155.2 mmHg. Incomplete cases were similar to complete cases in terms of age, sex, baseline SBP, and presence of diabetes and most other comorbidities; however, the participants with missing data had a higher probability of kidney disease and were more likely to be unemployed (Table S2).

**Table 1:** Baseline Characteristics of the ADAMPA Trial Patients (Complete Cases)

	Total (*N*=312)	Intervention (*N*=156)	Control (*N*=156)	*p* value
Men, *n* (%)	146 (46.8%)	70 (44.9%)	76 (48.7%)	0.50
Age, years, mean (SD)	64.4 (1.0)	64.8 (9.7)	63.9 (10.3)	0.40
Systolic blood pressure, mmHg, mean (SD)	155.2 (12.9)	155.2 (13.1)	155.2 (12.8)	0.96
Diastolic blood pressure, mmHg, mean (SD)	90.1 (8.0)	89.7 (7.9)	90.6 (8.1)	0.30
Body mass index, *n* (%)				
Normal (18–24 kg/m^2^)	48 (15.3%)	26 (16.7%)	22 (14.1%)	0.68
Overweight (25–30 kg/m^2^)	133 (42.6%)	63 (40.4%)	70 (44.9%)	
Obese (≥30 kg/m^2^)	130 (41.7%)	66 (42.3%)	64 (41.0%)	
Body mass index, mean (SD)	29.8 (4.9)	29.9 (5.2)	29.6 (4.7)	0.67
Level of education, *n* (%)				
No qualification	20 (6.4%)	13 (8.3%)	7 (4.5%)	0.53
Primary education	128 (41.0%)	64 (41.0%)	64 (41.0%)	
Secondary education	103 (33.0%)	51 (32.7%)	52 (33.3%)	
University degree or higher	61 (19.6%)	28 (18.0%)	33 (21.6%)	
Marital status, *n* (%)				
Single	21 (6.7%)	9 (5.8%)	12 (7.7%)	0.41
Married	216 (69.2%)	105 (67.3%)	111 (71.2%)	
Divorced	28 (9.0%)	18 (11.5%)	10 (6.4%)	
Widowed	47 (15.1%)	24 (15.4%)	23 (14.7%)	
Employment status, *n* (%)				
Permanent work	93 (29.8%)	43 (27.6%)	50 (32.1%)	0.19
Temporary work	6 (1.9%)	3 (1.9%)	3 (1.9%)	
Housewife	36 (11.5%)	17 (10.9%)	19 (12.2%)	
Unemployed	18 (5.8%)	14 (9.0%)	4 (2.5%)	
Pensioner	159 (51.0%)	79 (50.6%)	80 (51.3%)	
Smoking, *n* (%)	64 (20.5%)	28 (18.0%)	36 (23.1%)	0.26
Sedentarism, *n* (%)	138 (44.2%)	78 (50.0%)	60 (38.5%)	0.040
HRQoL (EQ5D), mean (SD)	0.85 (0.20)	0.85 (0.23)	0.86 (0.17)	0.48
Comorbidities, *n* (%)				
Diabetes	75 (24.0%)	38 (24.4%)	37 (23.7%)	0.90
Cerebrovascular disease	10 (3.2%)	4 (2.6%)	6 (3.9%)	0.52
Angina	2 (0.6%)	1 (0.6%)	1 (0.6%)	1.00
Acute myocardial infarction	4 (1.3%)	3 (1.9%)	1 (0.6%)	0.31
Peripheral artery disease	5 (1.6%)	2 (1.3%)	3 (1.9%)	0.65
Chronic kidney disease	18 (5.8%)	9 (5.8%)	9 (5.8%)	1.00
Years from onset of hypertension, mean (SD)	11.1 (9.3)	11.1 (9.6)	11.0 (9.0)	0.92
*N* antihypertensive drugs, mean (SD)	1.8 (0.9)	1.9 (0.9)	1.8 (0.9)	0.43
*N* concomitant treatments^a^, mean (SD)	2.35 (2.3)	2.25 (1.9)	2.5 (2.5)	0.43
Home blood pressure monitoring, *n* (%)	87 (27.9%)	38 (24.4%)	49 (31.4%)	0.17

*SD*, standard deviation; *HRQoL*, health-related quality of life; *EQ5D*, EuroQol 5D-3L

^a^In addition to antihypertensive medications

Between baseline and 12 months, BP values decreased in both groups, for both SBP (intervention: −19.0 mmHg; control: −16.1 mmHg) and DBP (intervention: −8.7 mmHg; control: −7.6 mmHg), resulting in an AMD in SBP at 12 months (main analysis) of −2.9 mmHg (95% CI: −5.9, 0.1, *p* = 0.061), and in an AMD in DBP of −1.9 mm Hg (95% CI: −3.7, 0.0, *p* = 0.052) (Table 2).

**Table 2:** Systolic and Diastolic Blood Pressure (mmHg), Crude and Adjusted, at Baseline and 12 Months’ Follow-up

	Blood pressure		Mean reduction from baseline to 12 months	Mean difference between groups: at 12 months
	Baseline	12 months		
*Systolic blood pressure*				
Intervention	155.2 (153.1, 157.2)	136.2 (134.0, 138.4)	−19.0 (−21.7, −16.2)	−3.0 (−6.2, 0.3) p = 0.071
Control	155.2 (153.2, 157.3)	139.2 (136.8, 141.6)	−16.1 (−18.5, −13.6)	
*Diastolic blood pressure*				
Intervention	89.7 (88.4, 90.9)	80.9 (79.5, 82.3)	−8.7 (−10.2, −7.2)	−2.1 (−4.2, 0.0) p = 0.049
Control	90.6 (89.3, 91.9)	83.0 (81.5, 84.6)	−7.6 (−9.0, −6.1)	
*Systolic blood pressure, adjusted*^*a*^				
Intervention		136.1 (135.2, 137.0)^b^		−2.9 (−5.9, 0.1) p = 0.061
Control		139.1 (138.3, 140.0)^b^		
*Diastolic blood pressure, adjusted*^*a*^				
Intervention		80.9 (80.2, 81.5)^b^		−1.9 (−3.7, 0.0) p = 0.052
Control		83.1 (82.4, 83.7)^b^		

^a^Adjusted for sex, age, baseline systolic blood pressure, obesity, diabetes (fixed effects) and general practitioner (random effect)

^b^Mean prediction from the fitted model

**Figure 2:**
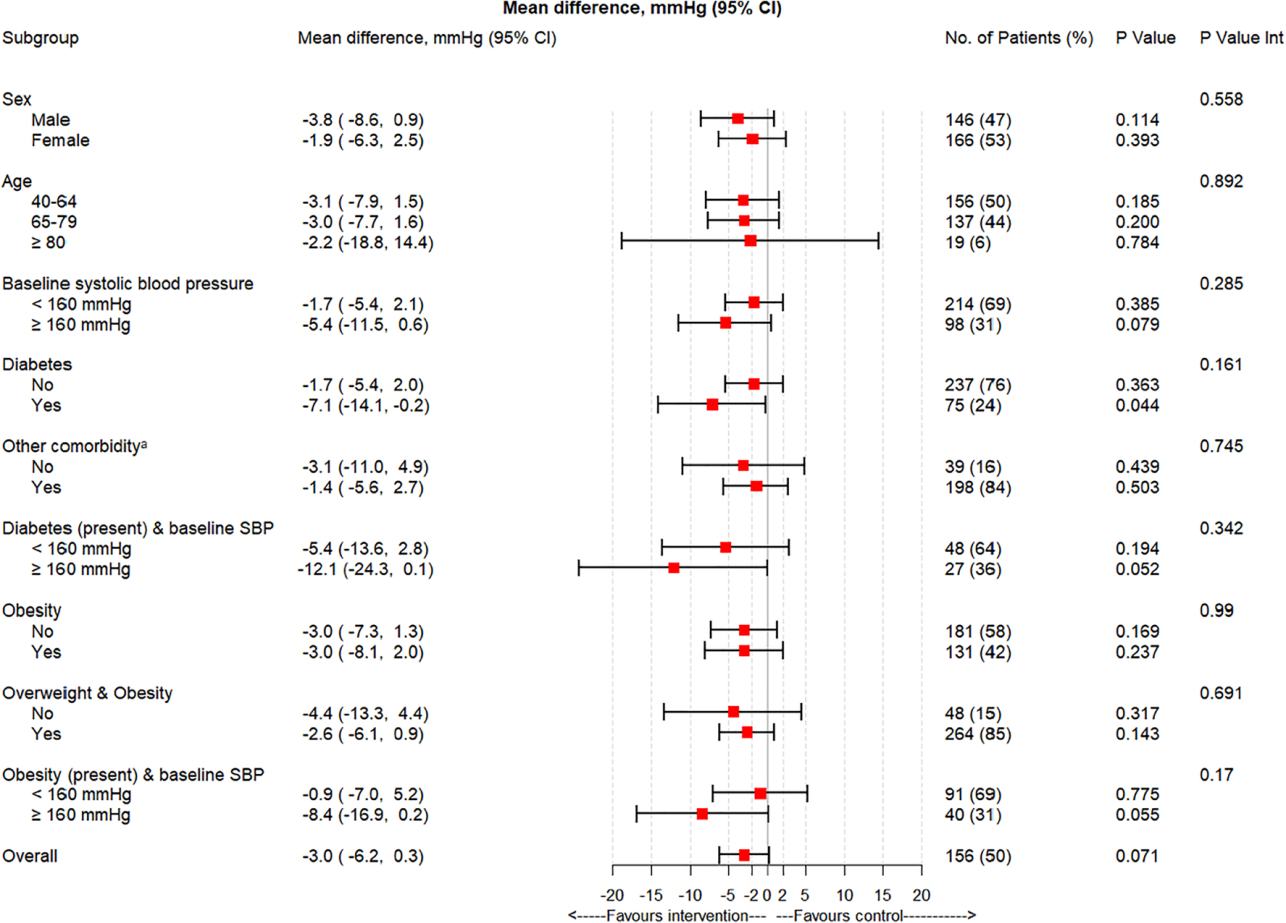
Mean difference in systolic blood pressure between intervention and control groups at 12 months’ follow-up by pre-specified subgroups. CI, confidence interval; SBP, systolic blood pressure. ^a^Other comorbidities include cerebrovascular disease, peripheral artery disease, chronic artery disease, chronic kidney disease, angina, and acute myocardial infarction.

The subgroup analysis (Fig. 2) suggested that the difference in SBP within pre-specified subgroups of the intervention and control arms was similar. However, the reduction was greater in patients with diabetes (MD −7.1 mmHg; 95% CI −14.1, −0.2; *p* = 0.044).

According to ESH/ESC guidelines establishing good control of BP at values under 140/90 mmHg (Table S1) (42), the percentage of patients who attained these targets at 12 months was 55.8% in the intervention group versus 42.3% in the control group (difference 13.5%; 95% CI 2.5%, 24.5%, *p* = 0.017). Applying the age-dependent recommendations, 12.8% of participants were within the target BP range in the intervention group, compared to 6.4% in the usual care group (difference 6.4%; 95% CI 0.0%, 12.9%; *p* = 0.055).

Regarding behavioral factors and quality of life (Table S3), no differences were observed for sedentarism, smoking, obesity, or EQ5D score.

There was an increase in antihypertensive drugs prescription in both groups at 12 months, but this was significantly more pronounced in the intervention group (Table S4), with an average of 0.24 more prescriptions (95% CI 0.03, 0.46; *p* = 0.027) compared to the control group. Fifty-eight percent of patients in the intervention group self-adjusted their medication at least once during the 12-month follow-up (either increasing doses or adding a new medication) (Table S5).

Regarding hypertension-related health services utilization (Table S6), no significant differences between groups were found over the 12-month follow-up. Only a few consultations were needed in addition to the protocolized follow-up visits, with no differences between groups.

Very few adverse events were reported during the follow-up, with no apparent differences between groups. Table 3 details those that could potentially be related to arterial hypertension, antihypertensive treatments, and/or the intervention, as well as serious adverse events, defined as any clinical event requiring hospitalization, endangering the patient’s life, or having an otherwise substantial impact on the patient’s health, as determined by the researcher.

**Table 3:** Adverse Events During the 12-Month Follow-up

	Intervention, *N*=156 *n* (%)	Control, *N*=156 *n* (%)
Adverse events potentially related to hypertension, antihypertensive treatment and/or intervention		
Syncope due to hypotension	2 (1.28%)	1 (0.64%)
Hypotension	3 (1.92%)	0 (0.00%)
Swelling of legs and/or ankles	0 (0.00%)	3 (1.92%)
Heart arrhythmia	1 (0.64%)	1 (0.64%)
Hospitalization due to hydro electrolytic disorder potentially associated with ARBs treatment	1 (0.64%)	0 (0.00%)
Heart palpitations	1 (0.64%)	0 (0.00%)
Transient ischemic attack	0 (0.00%)	1 (0.64%)
Serious adverse events^a^		
Breast cancer	2 (1.28%)	1 (0.64%)
Melanoma	0 (0.00%)	1 (0.64%)
Prostate cancer	0 (0.00%)	1 (0.64%)
Paroximal supraventricular tachycardia^b,c^	0 (0.00%)	1 (0.64%)
Acute pyelonephritis^b^	1 (0.64%)	0 (0.00%)
Hospitalization due to hydro electrolytic disorder potentially associated with ARBs treatment	1 (0.64%)	0 (0.00%)

^a^Defined as any clinical event requiring hospitalization, endangering the patient’s life, or having an otherwise substantial impact on the patient’s health, as determined by the researcher. These may be related or unrelated to hypertension, antihypertensive treatment, and/or intervention

^b^Adverse event that required hospitalization. *ARBs*, angiotensin receptor blockers

^c^This adverse event is also included under the category “Adverse events potentially related to hypertension, antihypertensive treatment and/or intervention”

## Discussion

The ADAMPA trial assessed the effectiveness of an intervention combining home blood pressure self-monitoring plus self-titration of antihypertensive medication (based on an individualized pre-arranged plan) and educational components versus usual care (also with educational components) in poorly controlled hypertensive patients. Our study did not show differences in the reduction of systolic blood pressure at 12 months (primary outcome) for the self-management intervention as compared to an educational-only intervention; however, the percentage of patients achieving good control at 12 months was higher in the intervention group compared to controls. Subgroup analyses for the primary outcome measure, though underpowered, showed consistent results, suggesting greater reductions in high-risk patients such as people with diabetes or with SBP above 160 mmHg. There was no evidence of between-group differences in adverse events, health services utilization, health-related quality of life, or behavioral changes, except for a reduction of the proportion of sedentary people in the intervention group (albeit with no differences with the control group at 12 months’ follow-up).

Several systematic reviews have assessed home blood pressure monitoring, although results have been heterogeneous, in part due to the combination with other co-interventions.^13, 17, 18, 40, 41^ In general, HBPM alone has little or no effect for lowering BP or improving control, but combined with other co-interventions it can lead to relevant BP reductions,^17, 29^ at least in the short term. The ADAMPA trial results are consistent with similar studies carried out in the UK National Health Service^23, 25, 26^ confirming that interventions combining HBPM, individualized BP targets, and medication self-titration are more effective than usual care for reducing SBP, even in different settings and with heterogeneous patients, doctors, and organizational schemes, and especially with different very degrees of patient empowerment. The absence of telemonitoring also differentiates the ADAMPA trial from the UK studies,^23, 25, 26^ where telemonitoring was used in all but one of the TASMINH4 trial arms,^23^ which showed similar BP reduction compared to the telemonitoring arm.

The absolute adjusted mean difference in BP (−2.9 mmHg for SBP and −1.9 mmHg for DBP) found in the ADAMPA trial falls in the lower range of similar studies,^23, 25, 26^ but these values still represent a relevant reduction in the risk of stroke and cardiovascular disease.^7^ In fact, a meta-analysis showed that similar blood pressure reductions (SBP −3.6 mmHg, DBP −2.4 mmHg) were associated with a 14% reduction in total cardiovascular events, a 28% reduction in strokes, and a 25% reduction in cardiovascular deaths after 5 years of follow-up.^42^ Moreover, these BP reductions entailed a higher increase in the percentage of patients achieving good control at 12 months in the intervention group compared to controls (55.8% vs 42.3%). And, importantly, these differences were achieved with no increase in adverse events, decrease in quality of life, or intensification of health services utilization (beyond the increase in antihypertensive medication). Beyond the aforementioned, it is key for an appropriate interpretation of our findings to note that the self-management intervention assessed in the present study differs from similar previous studies^23–27, 35^ in that it involved a high level of patient empowerment (mainly through self-adjustment of medication, without any kind of coaching/support), and was carried out in conditions of routine clinical practice, with no requirement of additional technological, human resources or health services use.

Because no other relevant changes were detected, the effect of self-monitoring may have been mediated by the intensification of antihypertensive medication, arising from doctors’ and patients’ sharpened awareness of individualized BP targets, the regular home monitoring of their attainment, and the self-adjustment of treatment in response to high BP values (in fact, additional to the medication changes made by the physicians as part of their routine clinical practice, 58% of patients self-adjusted their medication at least once without any additional contact with their family doctor). In this sense, the intervention would act mainly by reducing therapeutic inertia^43^ because of the patient’s more active role. Alternative (or complementary) explanations could include an increase in patient adherence (a potential effect of HBPM according to one meta-analysis^40^), changes in other variables not available in this study (such as salt or alcohol intake), or the reduction of sedentary behaviors.

Regarding secondary outcomes, we did not find differences between groups at 12 months in smoking, obesity, or sedentarism. We likewise found no differences in the use of health services, although this result is mediated by its context within a clinical trial with planned visits (for example, patients in the intervention group had to go to the practice in the following weeks after each treatment self-adjustment). In any case, and in addition to an extension of the follow-up to 24 months, we have planned qualitative studies (focus groups with doctors, nurses, and patients) and utilization studies (including aspects of inertia, adherence, and cost-effectiveness) based on data obtained from the electronic medical record, which may broaden our knowledge about the effectiveness, acceptability, and mechanisms of action of the intervention evaluated in our context.

Finally, the intervention was not associated with an increase in adverse events. Nevertheless, the frequency of hypotensive syncope seemed higher in the intervention group, although the extremely low figures do not allow for comparisons between groups. This should be further studied in larger trials.

The ADAMPA trial has some limitations. First, we had to stop recruiting patients prematurely for reasons unrelated to the study. Although the sample size obtained was sufficient to detect significant differences in the main analysis, the limited sample size reduces the accuracy of the estimates. Second, the ADAMPA trial is a non-masked study wherein both the patients and the research team knew the assigned group, enabling the presence of information biases such as the Hawthorne effect (patients modifying their behavior in response to their awareness of being observed), social desirability bias (patients overreporting positive behaviors or underreporting undesirable ones), and performance bias (physicians modifying their behavior). Third, throughout the study, doctors became familiar with the components of the intervention, and it is possible that they extended some of these components (e.g., fixing individualized BP targets) to the control group; this contamination bias would tend to skew the intervention effect towards the null. Fourth, the ADAMPA trial was underpowered to detect differences in clinical outcomes, but BP reduction is an excellent surrogate endpoint in hypertensive patients and is very well correlated with reductions in morbidity and cardiovascular mortality.^7, 44, 45^ Fifth, our study used strict inclusion criteria — for example, excluding correctly controlled hypertensive patients, who account for approximately half of the population with hypertension — and the generalization of its results to this patient population warrants caution.

## CONCLUSIONS

Self-management of blood pressure including home blood pressure monitoring, educational components, and patients’ self-titration of antihypertensive medication based on an individualized pre-arranged plan in the primary care setting may be a promising strategy for reducing blood pressure compared to usual care at 12 months of follow-up, without increasing healthcare utilization or adverse events. Our results suggest that, in the context of routine clinical practice, high-level patient empowerment strategies based on self-adjustment of antihypertensive treatments, with a pre-agreed plan and, without the need of additional medical visits (except in specific cases), the involvement of health professionals or health coaches, or the use of additional resources, may have relevant potential implications for both primary care practice and the health system as a whole.

## Supplementary Information

ESM 1 (DOCX 147 kb)

## Data Availability

The datasets used and/or analyzed during the current study are available from the corresponding author on reasonable request.
