# Supplementary material for: Home Blood Pressure Self-monitoring plus Self-titration of Antihypertensive Medication for Poorly Controlled Hypertension in Primary Care: the ADAMPA Randomized Clinical Trial
Source: J Gen Intern Med. 2022 Oct 11;38(1):81–9. doi: 10.1007/s11606-022-07791-z (PMC9849508; doi:10.1007/s11606-022-07791-z)
Supplement: Supplementary file 1 — (DOCX 147 kb) [file 11606_2022_7791_MOESM1_ESM.docx]

**Appendix**

**eMethods**: Exclusion criteria

1) Inability to self-manage their BP, for example due to dementia or significant cognitive impairment, at the discretion of the researcher performing the recruitment.

2) A history of orthostatic hypotension (fall > 20 mmHg from SBP after taking the orthostatic position)

3) SBP > 200 mmHg or DBP > 100 mmHg in the baseline examination,

4) Being prescribed more than four antihypertensive drugs,

5) Inclusion in another hypertension study or any clinical trial.

6) Presence of tremor or neurological disease that makes it difficult to perform HBPM,

7) Presence of arrhythmias,

8) Presence of terminal illness, chronic incapacitation to leave home or an acute cardiovascular event in the previous three months,

9) Hypertension managed directly by specialist doctors outside the primary care environment, 10) Pregnant women or those actively seeking to become pregnant,

11) Having a household member already enrolled in the study, and

12) Non- or temporary residents of Spain, due to limitations with follow-up.

**Table S1**. Blood pressure targets

| Age | SBP (mmHg) | DBP (mmHg) |
| --- | --- | --- |
| General recommendation | | |
| >18 years | <140 | <90 |
| If treatment is well tolerated | | |
| 18- 64 years^a^ | 120-129 | 70-79 |
| 65-79 years^a^ | 130-139 | 70-79 |
| ≥80 years^b^ | 130-139 | 70-79 |

SBP: systolic blood pressure; DBP: diastolic blood pressure

^a^Diabetic patients and patients with very high cardiovascular risk would potentially benefit the most in the lower interval of the recommended range.

^b^In frail patients, same recommended range as in 65-79 years if possible but with special attention to adverse effects of antihypertensive treatment and hypotension.

Source: 2018 European Guide for the management of Hypertension^27^

| **Table S2.** Baseline characteristics for patients with complete vs. incomplete follow-up | | | |
| --- | --- | --- | --- |
|  | **Complete cases (n=312)** | **Incomplete cases (n=45^a^)** | **p value** |
| Men, n (%) | 146 (46.8%) | 23 (51.1%) | 0.59 |
| Age, years, mean (SD) | 64.4 (10.0) | 65.4 (10.5) | 0.52 |
| Systolic blood pressure, mmHg, mean (SD) | 155.2 (12.9) | 156.6 (14.3) | 0.45 |
| Diastolic blood pressure, mmHg, mean (SD) | 90.1 (7.99) | 91.0 (7.90) | 0.50 |
| Body mass index, n (%) |  |  |  |
| Normal (18-24 kg/m^2^) | 48 (15.4%) | 6 (14.3%) | 0.92 |
| Overweight (25-30 kg/m^2^) | 133 (42.6%) | 17 (40.5%) |  |
| Obese (≥30 kg/m^2^) | 131 (41.9%) | 19 (45.2%) |  |
| Body mass index, mean (SD) | 29.8 (4.91) | 30.4 (5.70) | 0.45 |
| Level of education, n (%) |  |  |  |
| No qualification | 20 (6.4%) | 3 (8.6%) | 0.35 |
| Primary education | 128 (41.0%) | 19 (54.3%) |  |
| Secondary education | 103 (33.0%) | 7 (20.0%) |  |
| University degree or higher | 61 (19.6%) | 6 (17.1%) |  |
| Marital status, n (%) |  |  |  |
| Single | 21 (6.7%) | 4 (8.9%) | 0.27 |
| Married | 216 (69.2%) | 27 (60.0%) |  |
| Divorced | 28 (9.0%) | 8 (17.8%) |  |
| Widowed | 47 (15.1%) | 6 (13.3%) |  |
| Employment status, n (%) |  |  |  |
| Permanent work | 93 (29.8%) | 5 (14.3%) | 0.019 |
| Temporary work | 6 (1.9%) | 1 (2.9%) |  |
| Housewife | 36 (11.5%) | 1 (2.9%) |  |
| Unemployed | 18 (5.8%) | 6 (17.1%) |  |
| Pensioner | 159 (51.0%) | 22 (62.8%) |  |
| Smoking, n (%) | 64 (20.5%) | 11 (31.4%) | 0.14 |
| Sedentarism, n (%) | 138 (44.2%) | 13 (37.1%) | 0.42 |
| HRQoL (EQ5D), mean (SD) | 0.85 (0.20) | 0.82 (0.24) | 0.33 |
| Comorbidities, n (%) |  |  |  |
| Diabetes | 75 (24.0%) | 12 (26.7%) | 0.70 |
| Cerebrovascular disease | 10 (3.2%) | 1 (2.2%) | 0.72 |
| Angina | 2 (0.6%) | 1 (2.2%) | 0.28 |
| Acute myocardial infarction | 4 (1.3%) | 0 (0.0%) | 0.44 |
| Peripheral artery disease | 5 (1.6%) | 3 (6.7%) | 0.03 |
| Chronic kidney disease | 18 (5.8%) | 8 (17.8%) | 0.004 |
| Years of onset hypertension, mean (SD) | 11.1 (9.30) | 8.7 (7.8) | 0.15 |
| N antihypertensive drugs, mean (SD) | 1.7 (0.94) | 1.7(0.82) | 0.90 |
| N concomitant treatments^b^, mean (SD) | 2.35 (2.31) | 2.72 (1.64) | 0.40 |
| Home blood pressure monitoring, n (%) | 87 (27.9%) | 17 (50.0%) | 0.008 |
| SD: standard deviation; HRQoL: health-related quality of life; EQ5D: EuroQol 5D-3L.  ^a^Missing values: Body mass index: 3; Marital status: 10; Employment status: 10; Smoking: 10; Sedentarism:10; HRQoL (EQ5D):13; N antihypertensive drugs: 9; N concomitant treatments: 16; Years of onset hypertension: 11; Home blood pressure monitoring:11  ^b^In addition to antihypertensive medications | | | |

| **Table S3.** Behavioral risks and health-related quality of life at baseline versus 12 months’ follow-up | | | | |
| --- | --- | --- | --- | --- |
|  | **Baseline** | **12 months** | **Mean reduction from baseline**  **to 12 months** | **Mean difference**  **between groups at 12 months** |
| *Smoking, % (95% CI)* | | | | |
| Intervention | 18.0 (11.9, 24.0) | 16.0 (10.3, 21.8) | −1.9 (−10.3, 6.4) | −4.5 (−13.1, 4.1)  p=0.31 |
| Control | 23.1 (16.5, 29.7) | 20.5 (14.2, 26.9) | −2.6 (−11.7, 6.6) |  |
| *Sedentarism, % (95%CI)* | | | | |
| Intervention | 50.00 (42.2, 57.9) | 35.3 (27.7, 42.8) | −14.7 (−25.6, −3.9) | 2.6 (−7.9, 13.07)  p=0.63 |
| Control | 38.5 (30.8, 46.1) | 32.7 (25.3 40.1) | −5.77 (−16.4, 4.8) |  |
| *Obesity (BMI ≥ 30), % (95% CI)* | | | | |
| Intervention | 43.0 (35.2, 50.7) | 42.3 (34.6, 50.1) | −0.6 (−11.6, 10.3) | 0.6 (−10.3, 11.6)  p=0.91 |
| Control | 41.0 (33.3, 48.7) | 41.7 (33.9, 49.4) | 0.6 (−10.3, 11.6) |  |
| *Health-related quality of life (EQ5D), mean (95% CI)* | | | | |
| Intervention | 0.85 (0.81, 0.88) | 0.86 (0.83, 0.89) | −0.02 (−0.06, 0.03) | 0.01 (−0.03, 0.05)  p=0.61 |
| Control | 0.86 (0.83, 0.89) | 0.86 (0.83, 0.89) | −0.00 (−0.05, 0.04) |  |
| BMI: body mass index; CI: confidence interval | | | | |

| **Table S4.** Home blood pressure monitoring and medication changes between baseline and 12 months’ follow-up | | | | |
| --- | --- | --- | --- | --- |
|  | **Baseline** | **12 months** | **Mean change from baseline**  **to 12 months** | **Mean difference**  **between groups at 12 months** |
| *Home blood pressure monitoring, % (95% CI)* | | | | |
| Intervention | 22.4 (17.6, 31.1) | 96.8 (94.0, 99.6) | 72.4 (65.2, 79.7) | 52.6 (44.3, 60.8)  p<0.001 |
| Control | 31.4 (24.1, 38.7) | 44.2 (36.4, 52.0) | 12.8 (2.2, 23.5) |  |
| *Number of antihypertensive drugs, mean (95% CI)* | | | | |
| Intervention | 1.7 (1.6, 1.9) | 2.3 (2.2, 2.5) | 0.6 (0.5, 0.7) | 0.24 (0.03, 0.46)  p=0.027 |
| Control | 1.7 (1.6, 1.9) | 2.1 (1.9, 2.2) | 0.4 (0.3, 0.5) |  |
| *Number of concomitant treatments, mean (95% CI)* | | | | |
| Intervention | 2.7 (2.3, 3.0) | 3.0 (2.6, 3.4) | 0.34 (0.13, 0.55) | 0.24 (−0.11, 0.58)  p=0.18 |
| Control | 3.2 (2.8, 3.6) | 3.1 (2.7, 3.5) | 0.10 (−0.18, 0.38) |  |
| CI: confidence interval | | | | |

| Table S5. Self-titration of antihypertensive medication in the intervention group during the study period. | | |
| --- | --- | --- |
|  | **Patients with at least one treatment modification** | **Number of treatment modifications** |
|  | **n (%)** | **Mean (95%CI)** |
| Dose increased | 50 (32.05%) | 1.38 (1.22;1.54) |
| New medication added | 69 (44.23%) | 1.44 (1.20;1.69) |
| Any self-titration | 91 (58.33%)^a^ | 1.91 (1.61;2.16) |
| ^a^At least one treatment modification through self-titration (either an increase in dose or an addition of a new medication) | | |

**Table S6.** Health Services utilization during the 12-month follow-up

|  | **Total**  **(n=312)** | **Intervention (n=156)** | **Control (n=156)** | **p**  **value** |
| --- | --- | --- | --- | --- |
| Visits to the health center with appointment^a^, n (%) | | | | |
| 0-2 | 158 (50.6%) | 77 (49.4%) | 81 (51.9%) | 0.77 |
| 3 – 6 | 116 (37.2%) | 61 (39.1%) | 55 (35.3%) |  |
| 7 or more | 38 (12.2%) | 18 (11.5%) | 20 (12.8%) |  |
| Mean (SD) | 3.2 (2.6) | 3.2 (2.4) | 3.3 (2.9) | 0.78 |
| Visits to the health center without appointment^b^, n (%) | | | | |
| None | 272 (87.2%) | 137 (87.8%) | 135 (86.5%) | 0.94 |
| 1 | 27 (8.7%) | 13 (8.33%) | 14 (9.0%) |  |
| 2 or more | 13 (4.2%) | 6 (3.9%) | 7 (4.5%) |  |
| Mean (SD) | 0.19 (0.57) | 0.17 (0.52) | 0.21 (0.61) | 0.62 |
| Calls to the healthcare center, n (%) | | | | |
| None | 298 (95.5%) | 146 (93.6%) | 152 (97.4%) | 0.10 |
| 1 or more | 14 (4.5%) | 10 (6.4%) | 4 (2.6%) |  |
| Home visits, n (%) | | | | |
| None | 310 (99.4%) | 154 (98.7%) | 156 (100.0%) | 0.16 |
| 1 | 2 (0.6%) | 2 (1.3%) | 0 (0.0%) |  |
| Note: There were no emergency room visits or hospital admissions related to blood pressure.  ^a^Through previous request;  ^b^Non-delayable or urgent visits. | | | | |

**Figure S1.** Medication adjustment sheet

Figure S2. Instructions to patients

| **HOW TO ACT ACCORDING TO YOUR BLOOD PRESSURE MEASUREMENTS** | | | |
| --- | --- | --- | --- |
| **Remember:**   - Take your blood pressure twice a day, once in the morning and once in the evening, FOR the first seven days of each month. - Whenever your BP is taken, you should take it twice, waiting for 1-2 minutes in between. - Write down the lowest measure of both systolic blood pressure (TOP reading) measurements in your monthly notebook. - In the case of a strange value, or a possible error in a measurement, repeat it to get 2 valid measurements. Once you have entered your blood pressure values in your monthly notebook, act according to the following TABLE OF COLOURS OF ACTION, **unless your doctor has indicated otherwise**. | | | |
| **BLOOD PRESSURE VALUES**  **(in the morning or in the afternoon)** | | **READING** | **ACTION** |
| **VERY HIGH** | Your systolic blood pressure (TOP reading) is **180 or more**  or  Your diastolic blood pressure (BOTTOM reading) is **100 or more** | **Your blood pressure is too high** | **Contact your doctor or visit your health care centre.** |
| **HIGH** | If your systolic blood pressure (TOP reading) or your diastolic blood pressure (BOTTOM reading) is above the target values marked by your doctor. | **Your blood pressure is raised**  If **FOUR** or more readings a week, once a month were high, proceed with the medication change instructions. | **1^st^ step**: **Follow instructions provided by your doctor.**  **2^nd^ step: make an appointment with your doctor within 3 weeks after self-adjustment.** |
| **NORMAL** | Your blood pressure values are in the range of your BP target. | Your blood pressure is well controlled | Continue with your usual medication and when you go to visit your doctor for any reason, show your doctor your blood pressure booklet measurements. |
| **LOW** | Your systolic blood pressure (TOP reading) is **100 or less.** | **Your blood pressure is too low** | **Contact your doctor or visit your health care centre.** |
| **RED:** Contact your doctor or visit your health centre.  **YELLOW:** Proceed to self-adjustment at home with your doctor’s instructions and schedule an appointment for 3 weeks after self-adjustment. | | | |
| **IN CASE OF DOUBT, CONTACT YOUR GENERAL PRACTITIONER.** | | | |

Adapted and modified from: The Colour Coding Chart. Supplementary webappendix in: McManus RJ, Mant J, Bray EP, et al. Telemonitoring and self-management in the control of hypertension (TASMINH2): a randomised controlledtrial. Lancet 2010; published online July 8. DOI:10.1016/S0140-6736(10)60964-6.
